# Arterial Baroreceptor Reflex Counteracts Long-Term Blood Pressure Increase in the Rat Model of Renovascular Hypertension

**DOI:** 10.1371/journal.pone.0064788

**Published:** 2013-06-06

**Authors:** Vitaly A. Tsyrlin, Michael M. Galagudza, Nataly V. Kuzmenko, Michael G. Pliss, Nataly S. Rubanova, Yury I. Shcherbin

**Affiliations:** 1 Institute of Experimental Medicine, V. A. Almazov Federal Heart, Blood and Endocrinology Centre, St-Petersburg, Russian Federation; 2 Institute of Cardiovascular Disease, I. P. Pavlov Federal Medical University, St-Petersburg, Russian Federation; University of Southampton, United Kingdom

## Abstract

**Introduction:**

The present study tested the hypothesis that long-term effects of baroreceptor activation might contribute to the prevention of persistent arterial blood pressure (BP) increase in the rat model of renovascular hypertension (HTN).

**Methods:**

Repetitive arterial baroreflex (BR) testing was performed in normo- and hypertensive rats. The relationship between initial arterial BR sensitivity and severity of subsequently induced two-kidney one-clip (2K1C) renovascular HTN was studied in Wistar rats. Additionally, the time course of changes in systolic BP (SBP) and cardiac beat-to-beat (RR) interval was studied for 8 weeks after the induction of 2K1C renovascular HTN in the rats with and without sinoaortic denervation (SAD). In a separate experimental series, cervical sympathetic nerve activity (cSNA) was assessed in controls, 2K1C rats, WKY rats, and SHR.

**Results:**

The inverse correlation between arterial BR sensitivity and BP was observed in the hypertensive rats during repetitive arterial BR testing. The animals with greater initial arterial BR sensitivity developed lower BP values after renal artery clipping than those with lower initial arterial BR sensitivity. BP elevation during the first 8 weeks of renal artery clipping in 2K1C rats was associated with decreased sensitivity of arterial BR. Although SAD itself resulted only in greater BP variability but not in persistent BP rise, the subsequent renal artery clipping invariably resulted in the development of sustained HTN. The time to onset of HTN was found to be shorter in the rats with SAD than in those with intact baroreceptors. cSNA was significantly greater in the 2K1C rats than in controls.

**Conclusions:**

Arterial BR appears to be an important mechanism of long-term regulation of BP, and is believed to be involved in the prevention of BP rise in the rat model of renovascular HTN.

## Introduction

The role of arterial baroreceptor reflex (BR) in blood pressure (BP) regulation has been extensively studied for almost a century. Numerous studies in this field led to the introduction of the concept of BP self-regulation in the late 50s [1], [2]. This concept stems from the observations that any appreciable BP change results in the modulation of the activity of both aortic and carotid baroreceptors, with subsequent activation of arterial BR and restoration of the BP to the initial level.

However, the concept of BP self-regulation has been questioned by Guyton and colleagues, who suggested that long-term regulation of BP is ensured by the renal mechanisms, while the role of arterial BR is limited to the buffering of acute BP changes [3]–[6]. Currently, 2 groups of facts provide evidence against the involvement of arterial BR in the long-term BP regulation: (1) arterial baroreceptor denervation does not result in persistent BP rise and (2) baroreceptors rapidly adapt to the prevailing pressure, a phenomenon that is generally referred to as baroreceptor resetting. Indeed, denervation of baroreceptors in the carotid sinus and aortic arch in different animal species was associated with only transient elevation of BP followed by its normalization within a few days [4], [7]–[9]. Notably, however, this fact does not necessarily imply that arterial BR is not involved in the long-term regulation of BP. Alternatively, lack of persistent BP increase after baroreceptor denervation might be accounted for by the simultaneous denervation of chemoreceptors, which leads to hypoxemia and attenuation of BP rise [10]. It is also conceivable that the afferent signaling from both cardiac and cardiopulmonary receptors through the intact vagal fibers might, at least in part, compensate for the function of mechanoreceptor reflexes after carotid and aortic denervation. This notion is supported by the findings of studies on dogs [11], [12], which show that stable hypertension (HTN) can be induced only after denervation of all mechanoreceptive zones.

The phenomenon of baroreceptor resetting had been described in a landmark study by McCubbin et al. [13] on dogs with renal HTN. Later studies confirmed this observation [14], [15] and identified 2 different types of resetting: rapid (acute) baroreceptor resetting and the chronic one [16]. Baroreceptor resetting appears to be a major argument against the role of arterial BR in the long-term control of BP [3], [5], [6]. Despite the compelling evidence supporting the phenomenon of baroreceptor resetting, studies have shown that a high-salt diet resulted in the development of sustained HTN only in rabbits with genetic disruption of arterial BR, but not in the intact animals [17]. Acute intravenous administration of angiotensin II has been shown to cause baroinhibition of sympathetic nerve activity (SNA) (e.g., [18]). The findings related to the presence and time course of changes due to SNA baroinhibition under conditions of continuous angiotensin II infusion have been less consistent. For instance, Yoshimoto et al. [19] noted only a transient decrease in renal SNA and unchanged levels of lumbar SNA in angiotensin II-treated rats. In contrast, continuous infusion of angiotensin II over 7 days in the rabbits resulted in the sustained inhibition of renal SNA during the entire period of vasoconstrictor treatment despite baroreceptor resetting [20]. On the basis of these facts, arterial BR is considered one of the major modulators of sympathetic vasomotor tone in the long-term setting. For example, Osborn [21] recently highlighted the key role of the central neural, but not renal, mechanisms on the long-term control of BP. Thus, it seems reasonable to assume that the long-term effects of arterial BR activation might contribute to the prevention of persistent BP increase in experimental models of HTN. This hypothesis was tested experimentally in the present study.

The present study included 3 experimental series. The first series was aimed at evaluating arterial BR function and time course of BP changes after induction of 2K1C renovascular HTN, as well as the relationship between initial arterial BR sensitivity and severity of renovascular HTN. In Series 2, we assessed the effect of sinoaortic denervation (SAD) on the incidence and time to onset of 2K1C HTN. Further, cervical sympathetic nerve activity (cSNA) was assessed in normo- and hypertensive animals in Series 3.

## Methods

### Ethics Statement

All procedures were performed in accordance with the Guide for the Care and Use of Laboratory Animals, European Convention for the Protection of Vertebrate Animals used for Experimental and Other Scientific Purposes. The study protocol was approved by the local Ethics Committee. The experiments complied with the ARRIVE guidelines (http://www.nc3rs.org/ARRIVE).

### Animals

The experiments were performed on male Wistar, Wistar-Kyoto (WKY), and spontaneously hypertensive rats (SHR) aged 14–15 weeks and weighting 250–280 g. Our pilot experiments have demonstrated that sinoaortic denervation with or without subsequent renal artery clipping resulted in high mortality rates in animals under 14 weeks of age. Since hemodynamic measurements in all experiments were regularly performed during 8 weeks, all animals completed the protocol at the age of 22–23 weeks. Therefore, the comparisons were made between animals of the same age. The animals were maintained on a 12-h light/dark cycle and administered food and water *ad libitum*.

### Renovascular HTN Model

Two-kidney one-clip (2K1C) model of renovascular HTN was used throughout the studies. In brief, anesthesia was induced in male Wistar rats with sevoflurane (Abbott Laboratories Inc., USA) in oxygen followed by intraperitoneal administration of sodium oxybate at a dose of 1 g/kg. The left renal artery was exposed via midline laparotomy. A calibrated tantal clip with an internal diameter of 0.30 mm was placed around the artery. Then, the wound was closed in layers, and the animals were maintained for an additional 8 weeks.

### Arterial BR Testing

Under sevoflurane anesthesia, heparin-filled polyethylene catheters were introduced into the abdominal aorta (via the femoral artery) and femoral vein for invasive measurement of mean BP (MBP) and drug administration, respectively. The distal ends of both catheters were tunneled under the skin, exteriorized over the dorsal aspect of the neck, and secured to a skin suture. Three days after the operation and before the arterial BR testing, the exteriorized catheters were connected to the external tubing to ensure registration of MBP and phenylephrine administration. After that, the rats were allowed to adapt by placing them for at least 30–40 minutes in the home cage, with free access to water and food. MBP was measured by a miniaturized pressure transducer (Baxter, USA) and monitored on the computer using Chart software (AD instruments, Ltd., Australia). The RR interval duration determined in each animal by averaging 7 consecutive RR intervals in order to account for the respiratory modulation of heart rate. The arterial BR sensitivity was evaluated quantitatively by the change in RR interval induced by the increase in MBP caused by intravenous administration of phenylephrine (0.1–0.5 mg/kg) [22]. The coefficient of linear regression, expressing the ratio between changes in RR interval and MBP, was calculated [23], [24]. After registration of the initial values of MBP and RR interval, the sensitivity of arterial BR was tested 3 times with an interval of 5 min; the averaged value of arterial BR sensitivity was used for the final analysis.

### Series 1: Evaluation of SBP, RR Interval, and Arterial BR Sensitivity in the 2K1C Rats

**Figure 1:**
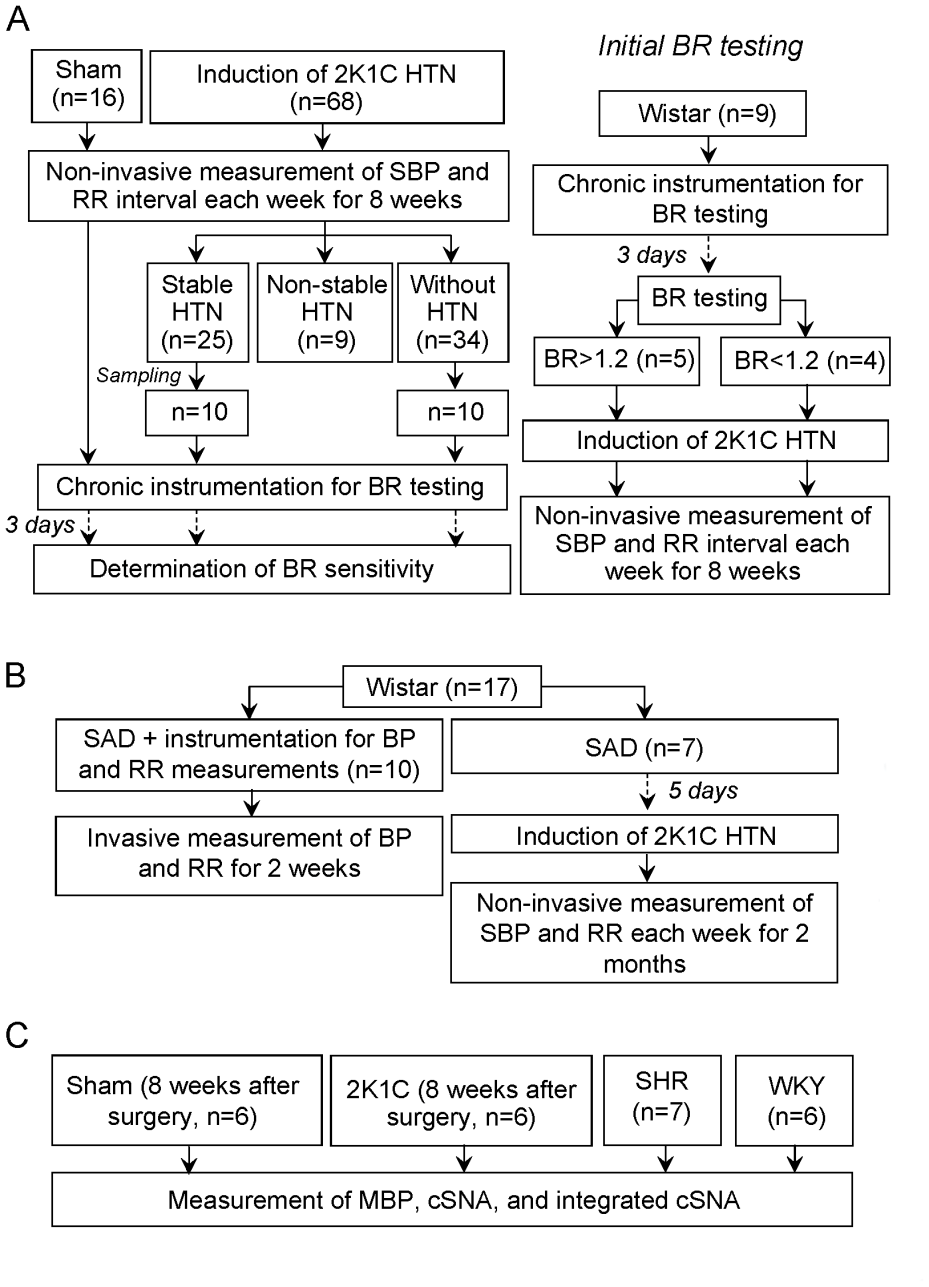
Experimental design. A: Series 1, evaluation of baroreflex function and time course of blood pressure changes after induction of two-kidney one-clip renovascular hypertension, as well as at investigation of the relationship between initial baroreflex sensitivity and severity of renovascular hypertension. B: Series 2, effect of sinoaortic denervation on the incidence and time to onset of two-kidney one-clip hypertension. C: Series 3, assessment of cervical sympathetic nerve activity in normo- and hypertensive animals. BP, blood pressure; BR, arterial baroreflex; cSNA, cervical sympathetic nerve activity; HTN, hypertension; 2K1C, two-kidney one-clip; MBP, mean blood pressure; RR, cardiac beat-to-beat interval; SAD, sinoaortic denervation; SBP, systolic blood pressure; SHR, spontaneously hypertensive rats; WKY, Wistar-Kyoto rats.

The following experimental groups were included in this Series (Fig. 1A): sham (n = 16), 2K1C (n = 68), and arterial BR +2K1C (n = 9) groups. (1) In the sham group, male Wistar rats were anesthetized, as described before, followed by midline laparotomy and isolation of the left renal artery without its clipping. After that, the wound was closed in layers, and the animals were allowed to recover. SBP and RR interval were non-invasively measured using a tail cuff with pulse sensor (ML125 NIBP controller, AD instruments Ltd., Australia) in all the 3 groups each week after sham surgery or induction of HTN for 8 weeks. The chart computer program (AD instruments Ltd., Australia) was used for data analysis. Eight weeks after surgery, the animals were re-anesthetized and chronically instrumented for the measurement of arterial BR sensitivity as described above. (2) In the 2K1C group, the procedures were generally the same as those used in the previous group, but the left renal artery was additionally clipped. According to the presence and stability of HTN, 8 weeks after surgery, the animals were divided into 3 subgroups: (a) those with stable HTN, (b) those with non-stable HTN, and (c) those without HTN (see *Results*). Arterial BR sensitivity was tested in the animals with stable HTN and without HTN. (3) The 3^rd^ group comprised rats subjected to initial testing of arterial BR +2K1C (n = 9); the anesthetized animals were chronically instrumented for measurement of BR sensitivity. Three days later, the baseline arterial BR sensitivity was determined as described in the section *“Arterial BR testing”.* Then, the rats were re-anesthetized, and 2K1C renovascular HTN was induced followed by 8 weeks of SBP and RR interval measurements. The animals with initial arterial BR testing were not tested again after HTN was established.

### Series 2: Effect of SAD on the Development of 2K1C Renovascular HTN

Bilateral SAD was performed in 17 anesthetized Wistar rats (Fig. 1B). Briefly, through a midcervical incision, the aortic nerves and cervical sympathetic nerve were identified and cut bilaterally with the aid of a surgical microscope. For the denervation of baroreceptors in the carotid sinus, the carotid bifurcation region was painted with 75% phenol solution. Denervation of baroreceptors was considered complete if BP elevation by 40–50 mm Hg was not associated with changes in RR interval. The animals were randomly allocated into 2 groups: (1) control (n = 10) group, in which SAD was followed by the catheterization of the aorta and femoral vein for chronic measurements of MBP and RR interval over 2 weeks; (2) SAD+2K1C (n = 7) group, in which 5 days after SAD, the animals were re-anesthetized, and a calibrated tantal clip with an internal diameter of 0.30 mm was placed around the left renal artery. The SBP and RR interval were non-invasively measured in the animals of the SAD+2K1C group every week after the induction of HTN for 2 months.

### Series 3: Assessment of cSNA in Normo- and Hypertensive Rats

Anesthesia was induced with diethyl ether, followed by intravenous infusion of alpha-chloralose at a dose of 45 mg/kg. The depth of anesthesia was judged from the absence of withdrawal reflexes and gross fluctuations of MBP and RR interval. The animals were mechanically ventilated (60 breaths/min), while myorelaxation was reached by pipecuronium bromide (Arduan, 1 mg/h). Core body temperature was maintained at 37.0±0.5°C by a feedback-controlled heating pad, and MBP was measured in the aorta. The RR interval was calculated online from the MBP signal. The left cervical sympathetic trunk was isolated under a surgical microscope, exposed, and cut at the level of superior cervical sympathetic ganglion. After that, the nerve was mounted on bipolar platinum hook electrodes in a pool of mineral oil. cSNA was obtained from the nerve electrode, amplified, and bandpass-filtered (10 Hz–2 kHz) by a preamplifier and fed into a nerve-traffic analyzer that provided the rectified, integrated (time constant, 0.01 s), and amplified (bandpass 40–600 Hz) nerve signal. According to the recommendations of Guild et al. [25], the integrated cSNA was expressed in absolute units. Both native and integrated cSNA were recorded for 300 s on a computerized recording and analyzing system with a sampling rate of 100 Hz for each channel. For calibration of integrated cSNA, the signal from the amplifier was processed in the same manner by using calibration option at the end of the experiment. The noise level observed in postmortem recordings was assumed to be zero level of the integrated activity. Two different types of analysis were used for the comparison of spontaneous cSNA values between the groups. Pulse-train analysis of the tonic integrated nerve activity was performed by means of a kick-sorting method “frequency tables.” For this purpose, the distribution of integrated cSNA magnitudes was expressed in percentages for each 300-s recording (STATISTICA 6.0 software). In each experimental group, the distribution of the magnitudes was averaged within each of 5 arbitrary ranges (2 to 5 µV; 5 to 10 µV; 10 to 15 µV; 15 to 20 µV; >20 µV). The values thus obtained are presented as “mean ± SEM.” For quantitative intergroup comparisons, integrated cSNA was expressed in µV×s. The integrated cSNA (µV×s) was determined using the following formula: cSNA = ∑ cSNA*_n_*/300, where *n* is the number of individual values of integrated cSNA registered during 300-s period. In each experiment, four 300-s recordings were made at four 5-min intervals. Four values of integrated cSNA were averaged to obtain the mean value. cSNA was analyzed in the following experimental groups (Fig. 1C): (1) sham-operated (n = 6) rats comprising normotensive Wistar rats that underwent isolation of the cervical sympathetic nerve with subsequent registration of cSNA; (2) 2K1C (n = 6) rats: cSNA was registered in the rats with stable 2K1C renovascular HTN 8 weeks after induction of renal artery stenosis; (3) SHR (n = 7); and (4) WKY (n = 6) rats.

### Statistical Analysis

Statistical analysis was performed using the STATISTICA 6.0 software package. The Kruskal-Wallis test was used to determine differences in cSNA, followed by pairwise inter-group comparisons performed using non-parametric Mann–Whitney *U* test. Differences in continuous data were tested by repeated-measures analysis of variance (ANOVA), followed by a Tukey post-hoc test. Relationship between BR sensitivity and SBP was assessed by the calculation of Pearson correlation coefficient and by multiple linear regression analysis. *p* values≤0.05 were considered significant. All data are presented as «mean ± standard error of mean».

## Results

### Series 1: SBP, RR Interval, and Arterial BR Sensitivity in 2K1C Rats

No difference was noted in the baseline (preoperative) SBP measured by the non-invasive method in the sham and 2K1C groups (125.9±7.7 and 124.6±8.1 mm Hg, respectively, *p*>0.05). Regular non-invasive measurements of SBP for 8 weeks after surgery showed that 34 of 68 rats did not develop HTN (Table 1, without HTN). Sustained elevation in SBP level after renal artery clipping was observed in 25 of 68 rats (Table 1, stable HTN). Further, 9 of 68 rats exhibited only a transient rise in SBP, which returned to the normal level 4–5 weeks after surgery (Table 1, non-stable HTN). The RR interval showed no difference in the animals with stable HTN and those without HTN.

**Table 1:** Systolic blood pressure and cardiac beat-to-beat interval over 8 weeks after left renal artery clipping in Wistar rats.

	Initial	Week 1	Week 2	Week 3	Week 4	Week 5	Week 6	Week 7	Week 8
Systolic blood pressure, mm Hg									
Sham (n = 16)	126.5±7.1	127.6±10.2	127.2±10.3	128.8±10.6	127.4±10.5	128.2±10.6	125.5±8.0	124.5±10.7	130.3±7.2
Stable hypertension (n = 25)	121.7±9.2	130.0±14.1	150.8±22.4*	174.4±21.5*	180.0±21.6*	178.4±24.9*	188.6±29.3*	181.4±26.2*	176.1±22.4*
Non-stable hypertension(n = 9)	127.3±6.1	136.2±13.2	145.8±19.7*	144.6±23.0	135.2±9.3	135.0±11.3	146.1±9.4*	143.2±10.5	129.3±6.2
Without hypertension (n = 34)	126.1±8.1	126.3±10.6	131.8±11.0	127.5±13.6	127.4±12.4	129.3±10.2	123.5±11.7	123,8±9,0	124.3±11.7
Beat-to-beat interval, ms									
Sham (n = 16)	159.4±15.2	158.7±16.1	159.8±14.4	159.5±10.3	163.0±12.8	159.4±11.9	173.2±13.4	177.6±10.7	178.5±13.2
Stable hypertension (n = 25)	164.6±18.1	164.5±15.6	160.8±12.3	155.5±12.5	165.4±15.5	164.7±17.6	166.4±14.0	166.6±16.3	168.2±16.3
Non-stable hypertension(n = 9)	172.7±15.2	173.2±23.5	168.1±19.2	163.4±21.4	182.5±15.2	181.7±23.7	177.8±25.4	176.3±17.3	184.2±13.4
Without hypertension (n = 34)	157.6±16.3	159.4±14.3	160.4±12.8	163.4±12.4	167.7±12.5	167.9±12.7	169.4±13.3	166.6±13.2	170.9±14.6

* *p*<0.05 versus controls.

**Figure 2:**
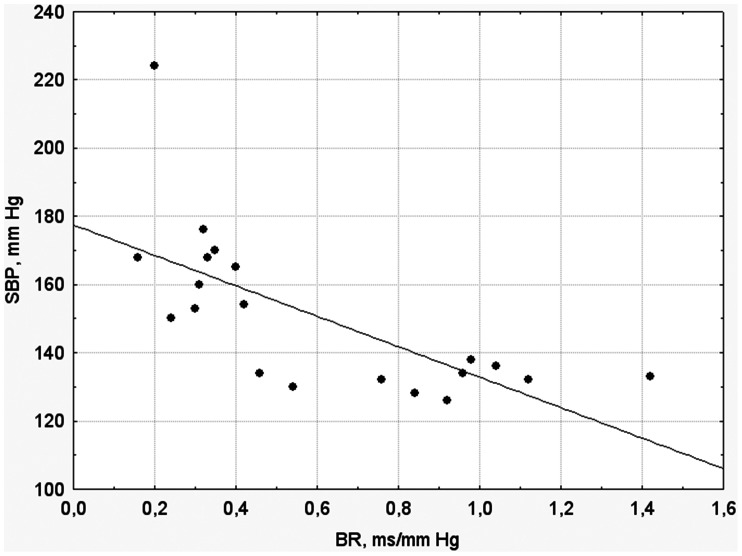
The relationship between baroreflex (BR) sensitivity and systolic blood pressure (SBP) in the pooled samples of Wistar rats with stable hypertension (n = 10) and without hypertension (n = 10) 8 weeks after induction of two-kidney one-clip renovascular hypertension. The inverse correlation between BR sensitivity and SBP is observed (r = 0.69, *p*<0.01).

Eight weeks after surgery, 20 animals were randomly chosen from the groups with stable HTN and without HTN for chronic instrumentation and arterial BR testing. 10 animals were sampled from stable HTN cohort and 10 more animals from the group without HTN. We considered unnecessary to instrumentalize all animals (n = 59) since, according to our preliminary estimates, the sample size of 20 would be enough to get a statistically meaningful result. Arterial BR sensitivity in the animals with stable HTN and without HTN averaged 0.35±0.12 and 0.78±0.18 ms/mm Hg, respectively (*p*<0.05). Notably, arterial BR sensitivity in the rats without HTN was similar to that in sham-operated animals (0.81±0.21 ms/mm Hg). Fig. 2 illustrates the presence of a significant inverse correlation between arterial BR sensitivity and SBP in the pooled sample of animals consisting of 10 rats each with stable HTN and without HTN (r = 0.69, *p*<0.01).

According to the results of the initial arterial BR testing performed before the establishment of 2K1C HTN, the animals were divided into 2 subgroups: (1) those with arterial BR sensitivity of <1,2 ms/mm Hg (0.87±0.08 ms/mm Hg, n = 4) and (2) those with arterial BR sensitivity of >1,2 ms/mm Hg (1.46±0.11 ms/mm Hg, n = 5). An inverse relation between initial arterial BR sensitivity and BP was observed in the 2K1C rats from the 3^rd^ week after renal artery stenosis onwards. In particular, at the 8^th^ week after surgery, SBP and MBP were 197±22.9 and 169±13.3 mm Hg in rats, respectively, with initial arterial BR sensitivity less than 1,2 ms/mm Hg and significantly lower (160±16.7 and 142±11.4 mm Hg, respectively, *p*<0.05) in rats with high arterial BR sensitivity (>1,2 ms/mm Hg). Thus, the animals with greater initial arterial BR sensitivity tended to have lower BP values after renal artery clipping than those with lower initial arterial BR sensitivity.

### Series 2: Effect of SAD on the Development of 2K1C Renovascular HTN

In controls (n = 10), SAD caused significant MBP elevation and decrease in RR interval for 2 days after surgery. Thereafter, the MBP values decreased, while the increased heart rate persisted for the entire period of observation. Three days after SAD, MBP averaged 108±24.2 mm Hg and was similar to its initial value.

**Figure 3:**
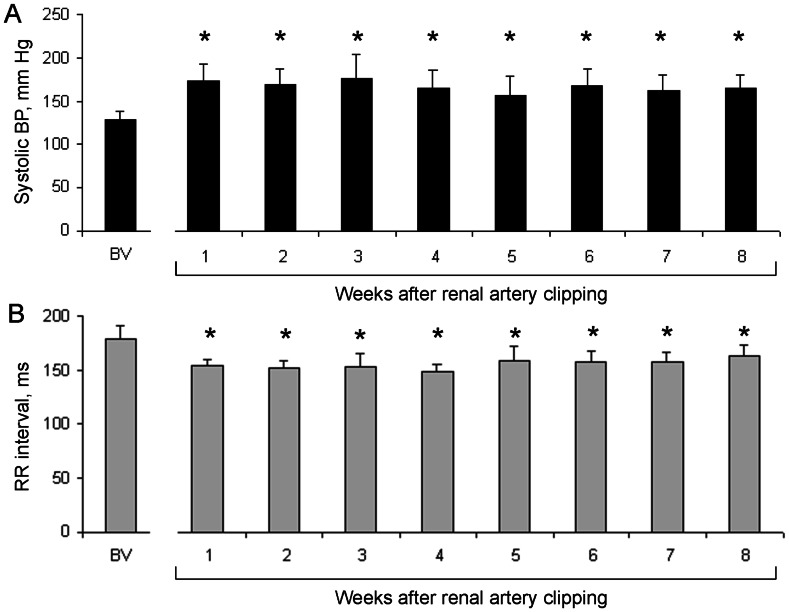
Hemodynamic parameters in the animals with sinoaortic denervation. A: Systolic blood pressure (SBP) and B: Cardiac beat-to-beat (RR) interval before and after renal artery clipping in the rats with sinoaortic denervation. BV, baseline value; BP, blood pressure. *p<0.05 versus baseline.

In the animals of SAD+2K1C group (n = 7), SBP averaged 128±10.2 mm Hg before renal artery clipping. However, renal artery clipping in these animals invariably resulted in both significant SBP elevation and RR interval decrease, which became evident within 1 week of renal artery clipping (Fig. 3). In contrast, 2K1C rats with stable HTN in the previous Series developed significant BP elevation only 2 weeks after renal artery stenosis. Eight weeks after renal artery clipping, SBP in the animals of the SAD +2K1C group averaged 164±14.9.

### Series 3: cSNA in Normo- and Hypertensive Rats

**Figure 4:**
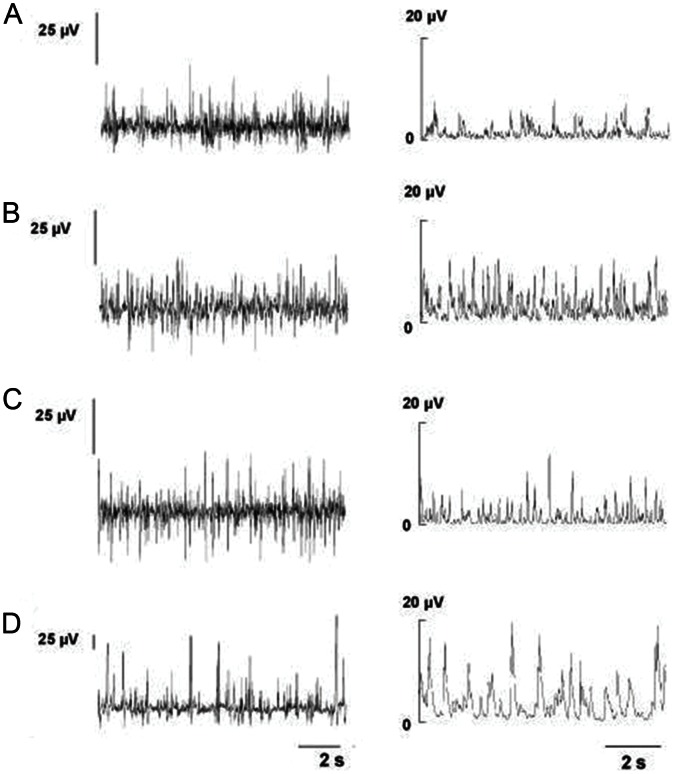
Cervical sympathetic nerve activity (left) and integrated electrical activity of cervical sympathetic nerve (right). A: Normotensive Wistar rats (n = 6). B: Wistar rats with two-kidney one-clip renovascular hypertension (n = 6). C: Wistar-Kyoto rats (n = 6). D: Spontaneously hypertensive rats (n = 7).

Integrated spontaneous cSNA in normotensive Wistar rats averaged 98.7±22.07 µV×s. This parameter was significantly higher in the animals with 2K1C HTN (191.5±29.31 µV×s, *p*<0.05 versus sham-operated rats, Fig. 4). Integrated spontaneous cSNA in the WKY and SHR rats was 181.6±46.67 and 479.0±84.93 µV×s, respectively (*p*<0.05). Amplitude analysis of cSNA showed that low-amplitude (2–5 µV) electrical activity appeared to be most prevalent in both normo- and hypertensive rats. However, the relative contribution of mid- (5 to 10 µV) and high-amplitude (>10 µV) electrical activity was greater in hypertensive rats than in normotensive ones. For example, the contribution of mid- and high-amplitude activity to the total cSNA in 2K1C group was, respectively, 3 and 1.5 times higher than that in the sham-treated animals (Table 2). Moreover, the contribution of mid- and high-amplitude activity was significantly higher in SHR than in the WKY rats, thereby reflecting the greater sympathetic activation in the former (Table 2).

**Table 2:** The relative contribution of low-, mid-, and high-amplitude integrated activity in the cervical sympathetic nerve (in percentage to total electrical activity) in the experimental groups.

	2< µV<5	5≤ µV<10	10≤ µV <15	15≤ µV <20	≥20 µV
Sham (n = 6)	93.7±2.3	5.3±1.8	0.8±0.4	0.2±0.1	0
2K1C (n = 6)	83.1±7.8	15.4±4.2	1.5±0.6	0	0
WKY (n = 6)	93.1±3.6	6.5±3.4	0.4±0.2	0	0
SHR (n = 7)	59.1±10.4	21.7±3.2	11.4±4.1	4.6±2.7	3.2±2.2

2K1C – Wistar rats with 2-kidney 1-clip renovascular hypertension; WKY – Wistar-Kyoto rats; SHR – spontaneously hypertensive rats.

## Discussion

The results of the present study showed that BP elevation in the 2K1C HTN model was associated with significant MBP variability, which was comparable to that in the animals with impaired arterial BR sensitivity. Further, we found that SBP elevation in the rats with 2K1C HTN was associated with decreased sensitivity of arterial BR (Series 2; Fig. 2).

Previous studies have shown that arterial BR sensitivity can differ significantly throughout the day, both in animals and normotensive humans [26], [27]. Further, a clear relationship between initial MBP level, the magnitude of its rise in response to administration of standard dose of norepinephrine, and arterial BR sensitivity has been established. In particular, minimal arterial BR sensitivity coincided with the maximal plasma concentration of norepinephrine [26]. It is well documented clinically that arterial BR sensitivity is decreased even at the very early stages of essential HTN [28], [29]. This decrease in arterial BR sensitivity in HTN patients is believed to be centrally mediated [28], [29].

SAD-induced BP elevation is often referred to as neurogenic HTN [1]. The development of neurogenic HTN after SAD is considered one of the major arguments in favor of the involvement of arterial baroreceptors and sympathetic nervous system in the long-term control of BP [30]. However, recent studies have clearly shown that chronic SAD does not result in persistent BP elevation [3]–[6], thereby suggesting that arterial BR function is aimed exclusively at buffering the acute BP changes. Some published data are inconsistent with this concept. For example, Weinstock and Borosh [31] demonstrated that increased salt intake in the normotensive rabbits with initially low arterial BR sensitivity resulted in sustained BP elevation, while animals with initially high arterial BR sensitivity did not develop HTN. Further, renal denervation has been shown to prevent the development of salt-sensitive HTN even in the rabbits with low arterial BR sensitivity. In addition, catheter-based renal sympathetic denervation is currently considered a promising therapeutic approach in patients with severe drug-resistant HTN [32].

Several factors are generally believed to contribute to the pathogenesis of 2K1C renovascular HTN. One of the major factors leading to BP elevation after renal artery clipping is increased renin release from the juxtaglomerular cells with subsequent increase in the plasma angiotensin II concentration. The mechanisms of angiotensin II-induced BP rise include vascular smooth muscle cell contraction caused by the direct stimulation of angiotensin II type 1A receptors, structural changes of the arterial wall, and activation of the sympathetic nervous system [33]. Our data on the increased values of cSNA in the animals with sustained renovascular HTN as well as in the SHR (Series 3; Fig. 4, Table 2) are in good agreement with the latter mechanism. Further, increased sympathetic tone is thought to play a key role in the development of renovascular HTN [34]. The increased activity of the sympathetic nervous system in renovascular HTN might be, at least partly, explained by the altered afferent input from the ischemic kidney since both dorsal root nerve lesion at T_8_–L_2_ and denervation of the ischemic (but not intact) kidney prevented the development of renovascular HTN [35], [36]. On the basis of the abovementioned experimental evidence, 2 assumptions on the involvement of arterial BR in long-term control of BP can be introduced. First, if arterial BR is indeed involved in the long-term BP control, then the animals with low baseline arterial BR sensitivity should be more susceptible to renovascular HTN than those with high BR sensitivity. Second, provided that arterial BR is responsible for long-term regulation of BP, both the incidence and time to onset of HTN should be greater in the 2K1C animals with BR dysfunction than in those with normal BR function. The data obtained in the present study confirmed both predictions. The results of our experimental Series 1 indicate that the initial arterial BR sensitivity largely determined the magnitude of BP elevation in the rat model of renovascular HTN. Although SAD itself resulted only in increased BP variability but not in persistent BP rise, the subsequent renal artery clipping invariably resulted in the development of sustained HTN (Series 2; Fig. 3). Notably, the time to onset of HTN was found to be shorter in the rats with impaired arterial BR function than in those with intact baroreceptors.

The present study has several methodological limitations. First, the relationship between preexisting arterial BR sensitivity and the eventual development of HTN would be more convincing if the numbers of animals in this Series were greater. However, the induction of 2K1C HTN in chronically instrumented rats and its subsequent maintenance for 8 weeks is technically challenging. Therefore, only 9 rats were included in this analysis. Second, the measurements of cSNA might have been confounded by anesthesia. Third, it would be interesting to compare the relationship between BR sensitivity and cSNA in chronically instrumented 2K1C rats. Lastly, the mechanisms of arterial BR involvement in the attenuation of renovascular HTN were not investigated in this study. Future studies will address these important issues.

In conclusion, our findings cast doubt on the notion that arterial BR is involved in long-term control of BP [3]–[6], [37]. Our results are consistent with those of studies showing that renovascular HTN develops because of concurrent arterial BR inhibition and renin-angiotensin system activation [38]–[40]. Further, the activity of renal sympathetic nerves responsible for the regulation of sodium excretion by the kidney seems to be, at least partly, modulated by the long-term effects of arterial BR. Arterial BR appears to be an important mechanism of long-term regulation of BP by playing a role in the prevention of BP rise in the rat model of renovascular HTN.
